# Sciducio: a practical framework for guiding the development and leadership of the academic research environment

**DOI:** 10.3389/frma.2023.1205874

**Published:** 2023-10-09

**Authors:** Lee Stoner, Craig Paterson, Lane Perry, Simon Higgins, Nathan Woolard, Patricia Pagan Lassalle, Emma Cowley, Yolanda Lassalle

**Affiliations:** ^1^Department of Exercise and Sport Science, University of North Carolina at Chapel Hill, Chapel Hill, NC, United States; ^2^Department of Epidemiology, The Gillings School of Global Public Health, University of North Carolina at Chapel Hill, Chapel Hill, NC, United States; ^3^Center for Health Promotion and Disease Prevention, University of North Carolina at Chapel Hill, Chapel Hill, NC, United States; ^4^School of Marketing, Entrepreneurship, Sports Management, and Hospitality and Tourism, College of Business, Western Carolina University, Cullowhee, NC, United States; ^5^Collins College of Business, University of Tulsa, Tulsa, OK, United States; ^6^LaSalle Group LLC, Raleigh, NC, United States

**Keywords:** laboratory, academia, management, planning, mentorship, Business Model Canvas, Lean Canvas

## Abstract

The goal of this paper is to introduce Sciducio, a practical framework for guiding the development and leadership of an academic research environment. The principal audience for this framework is new academics, that is individuals beginning a tenure-track position in the U.S or a lecturing position elsewhere in the world. However, we also believe this framework will be of use to established academics searching for structure, academics moving to a new institution, and can serve as a training tool for doctoral and postdoctoral mentees. We briefly describe the theory supporting Sciducio, outline the framework and its individual components (blocks), then provide suggested instructions for use. We provide suggested instructions (i.e., descriptive rather than prescriptive), because there is no one-size-fits-all approach for ensuring success. Sciducio incorporates three domains (Plan, Manage, and Deliver), encompasses eight blocks, and is intended to fit on one-sheet of paper or one screen. The Plan domain includes the blocks: value, strategy, and leadership. The Manage domain includes the blocks: activities, key resources, and finances. The Deliver domain includes the blocks: solution and channels. Considering each of the framework blocks is complex, we cannot provide full justice to each component. This paper serves as a general overview and subsequent papers will be more topic specific. Additionally, we encourage others to contribute to and advance this framework.

## Introduction

**Figure 1:**
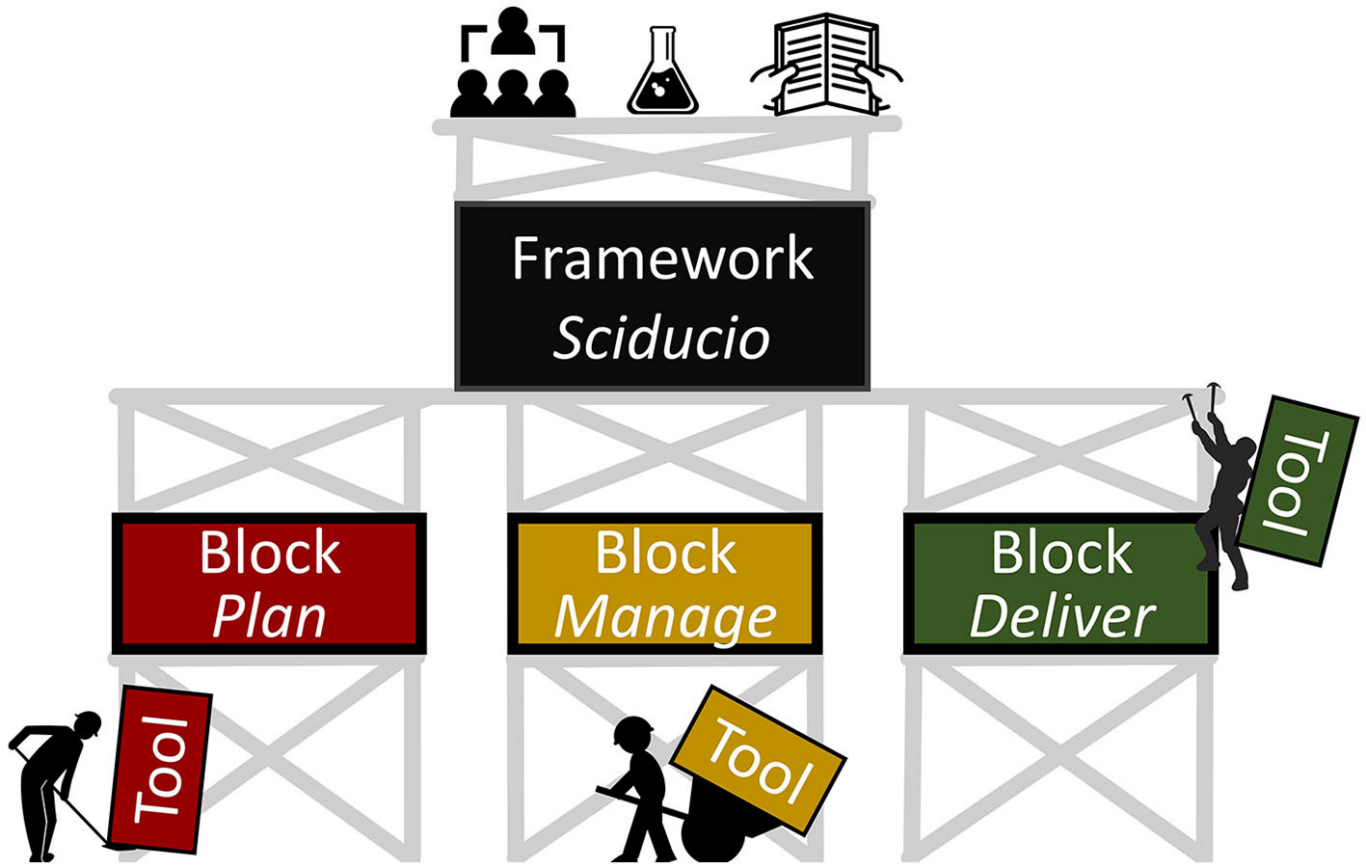
Conceptual overview of Sciducio. The Sciducio framework consists of several building blocks, which serve as the scaffolding to the framework. A block is latent construct and is descriptive rather than prescriptive. Tools are required to develop and apply the blocks.

The goal of this paper is to introduce Sciducio (phonetic pronunciation: sai-du-she-oh), a term coined by combining the Latin noun for science (scientia) and verbs for lead (ducat) and develop (adduco). The intent of Sciducio is to provide a practical framework for guiding the development and leadership of an academic research environment. As depicted in Figure 1, the framework (Sciducio) consists of a number of *blocks*, which are the scaffolding to the framework. A block is a latent construct and is descriptive rather than prescriptive. *Tools* (which we use synonymously with *strategies)* are required to develop and apply the blocks. The principal audience for this framework is new academics faced with the daunting task of establishing their research agenda—which most commonly occurs without a manual! However, Sciducio will also benefit:

- Academics moving to a new institution.
- Academics remaining at their existing institutions, who could use a beacon to manage the diverse and ever evolving components of maintaining a research agenda and research teams.
- Doctoral and postdoctoral mentees, i.e., as a training tool.

Below, we briefly describe the theory supporting Sciducio, outline the framework and its individual components (block), then provide suggested instructions for use. The word suggested is emphasized as the intent of Sciducio is to be descriptive rather than prescriptive—there is no singular path to success! It must be acknowledged that each of the blocks comprising Sciducio are complex and incorporate several existing models and concepts. With Sciducio, we have attempted to provide one comprehensive yet simple and practical framework. However, considering the complexity of each block, we cannot provide full justice to each. As such, this paper serves as a general overview and subsequent papers from our team will be more topic specific. We also encourage contributions from other academics. Contributions may be made in multiple capacities, including commentaries that suggest edits or additions to a block, highlighting specific tools to help individuals work through Sciducio, or discussing how Sciducio was adapted for use in specific environments.

Throughout this paper we bold structural (e.g., heading, signposting) terms, Capitalize Existing Frameworks/Models, *italicize key concepts*, and underline points of emphasis. Definitions for key terms, concepts and models are provided in Table 1.

**Table 1:** Description of key terms and concepts supporting the development of Sciducio.

**Item**	**Category**	**Description**
Block	Term	Actionable component of Sciducio, to *build* plan
Business model canvas	Model	1-sheet, strategic business model template
Channels	Concept	Avenues for delivering value proposition, e.g., Publications
Decision-making environment	Concept	Considers nature of problem, risk, and culture
Domain	Term	Conceptual area of influence, to group blocks
Entrepreneurial mindset	Model	Identifies opportunities and learns from setbacks
Entrepreneurship	Term	Individual that acts on new, disruptive idea
Fixed costs	Concept	Unchanged across applications
Focus area	Concept	High-priority areas when working toward the vision
Framework	Concept	Simplified system for the realization of defined goal(s)
Idealized influence	Concept	Modeling desirable behavior and passion toward goals
Impact	Concept	Anticipated impact of delivering the solution
Individual consideration	Concept	Shared vision and trust in employees
Inspirational motivation	Concept	A vision to foster a strong sense of purpose and expectations
Intellectual stimulation	Concept	Focus on new experiences, opportunities and innovative thinking
Key activities	Concept	Most important activities when executing value proposition
Key performance indicators	Concept	Measurable values to indicate whether objectives met
Key resources	Concept	Resources required to execute plan
Leadership	Term	Visionary, mentor, and source of inspiration for innovation
Lean canvas	Model	Evolved from Business Model Canvas, organized in logical order
Mindset	Term	Interrelated beliefs, assumptions, and knowledge
Mission statement	Concept	The main function of the organization
Objectives	Concept	Specific, measurable targets that align with focus area(s)
Organizational culture	Concept	Shared values, beliefs and assumptions.
Partner networks	Concept	Strategic alliances
Problem	Concept	The problem that will be addressed
Projects	Concept	Specific activities that will help to achieve the objectives
Revenue streams	Concept	Income generated to support fixed and variable costs
Sciducio	Model	Practical development and leadership framework
Solution	Concept	The intended final product
Target audience	Concept	Population to which solving the problem will be beneficial
Tool	Term	A resource to help plan and complete a block
Transformational leader	Model	Inspires positive change
Unfair advantage	Concept	Competitive edge or point or difference
Value proposition	Concept	Vision of solution
Value statement	Concept	Expected behaviors within the organization
Variable costs	Concept	Costs associated with specific key activities
Vision statement	Concept	Vision for the future state of the organization

Cross-referenced terms are bolded.

## Theoretical overview

Our goal with Sciducio was/is to create a singular, digestible framework that assists in guiding the development and leadership of an academic research environment. To assist with this goal, we reviewed several existing resources (e.g., frameworks, theories, tools) that have been successfully utilized within industry. The need for exploring and assimilating multiple resources was based on two assumptions: (i) there is no one-size-fits-all approach for both developing and leading a new business (or academic research environment); and (ii) most academics do not have business degrees and have neither the time or inclination to comprehend and utilize multiple, disparate resources that can assist with purpose, project, and people management.

The resources we reviewed are categorized and presented below, along with rationale pertaining to how and why these resources guided the development of Sciducio. Considering the focus of this paper is to introduce Sciducio, we acknowledge that we were unable to fully rationalize how and why each resource was selected. We did, however, carefully select references to support each resource; said references will be useful for those interested in deeper learning. Importantly, each reference used to support a concept is a seminal reference in the fields of entrepreneurship (startup development) and project management.

In summary, our goal is to provide enough insight into the underpinnings of Sciducio for it to make sense, but not so much that it is perceived as a finalized version of the conceptual framework. Our goal is to have the framework developed further through topic-specific papers (including works from external parties), collaboration, and critique.

### Models

In developing Sciducio, we began by exploring business models that could be adapted and serve as a foundation. A business model specifically describes how an organization *creates, delivers*, and *captures* value (Olsen, 2015). These verbs lend themselves well to the academic research environment. We identified two widely used models: (i) Business Model Canvas (Osterwalder and Pigneur, 2010), and (ii) Lean Canvas (Maurya, 2022). The Business Model Canvas, a widely used tool for conceiving the business model of a startup, is comprised of five domains made up of nine building blocks: infrastructure (*key activities, key resources*, and *partner network*), offering (*value propositions*), customers (*customer segments, channels*, and *customer relationships*), finances (*cost structure*), and revenue streams. This model is intended to be printed on a large canvas, to which groups of individuals can contribute. The Lean Canvas evolved from the Business Model Canvas and is similarly comprised of nine building blocks (problem, customer segments, unique value proposition, solution, channels, revenue streams, cost structure, key metrics, and unfair advantage). However, the block titles and purposes sit in a logical order, beginning with the problem (*value proposition*). Ultimately, both models have been conceived to translate thoughts into assertive, actionable language, and to do so with minimal time demand.

### Strategic plan

The models discussed above created an excellent foundation for Sciducio but do not consider *strategy* and have been criticized for not being process-oriented, and minimizing external influences of environment, market, and other forces. Our goal with Sciducio was to create a singular, digestible framework. One that will assist with turning a plan into action, monitoring performance, considering external forces, and ensuring continuous reflection and growth. A *strategic plan* is used to help companies focus their effort, typically when transitioning the organization toward a new direction (Viardot, 2017). A *strategic plan* may include: (i) a *mission statement*, i.e., the main function of the organization; (ii) a *vision statement*, i.e., the vision for the future state of the company; (iii) a *value statement*, i.e., how individuals should behave when representing the organization and shaping culture; (iv) *focus areas*, i.e., high-priority areas when working toward the vision; (v) *objectives*, i.e., specific, measurable targets that align with one or more focus area; (vi) *projects*, i.e., specific activities that will help to achieve the objectives; (vii) *key performance indicators (KPIs)*, i.e., measurable values to indicate whether the objectives are being met; and (viii) *environmental scan or SWOT Analysis*, i.e., externally focused analysis of conditions supporting or stymieing business focus. The KPI concept, which aligns with the *key metrics* block of the Lean Canvas (Maurya, 2022), refers to how performance is monitored (e.g., X subscriptions, X units sold, X downloads).

### Leadership

A good leader is equal parts visionary, mentor, and source of inspiration for innovation, and accomplishes this by creating a distinctive team *culture* and *mindset* (Northouse, 2021). The Business Model Canvas (Osterwalder and Pigneur, 2010) or Lean Canvas (Maurya, 2022) do not overtly consider leadership. Rather, leadership is typically fostered through formal education, training, and independent learning. We are incorporating leadership within Sciducio because it is not typically a requirement of science doctorate training and may not be a concept that is formally considered as one develops a research team and environment. We encourage leadership to be considered during the planning phase of Sciducio because it is paramount to morale, resiliency, success, and sustainability (Groysbrg et al., 2018; Maestre, 2019; Seals, 2021, 2022a).

To guide this component of Sciducio, we primarily took inspiration from S*ervant Leadership* (Robert, 2022), the *5 Levels of Leadership* (John, 2011), and particularly the *Four I's of Transformational Leadership* (Northouse, 2021). The four i's encompasses: (i) *intellectual stimulation*, i.e., emphasizing new experiences, new opportunities and innovative ways of thinking; (ii) *individual consideration*, i.e., communicating a clear and shared vision and trusting employees to make decisions and complete tasks within their defined areas; (iii) *inspirational motivation*, i.e., for communicating a vision to foster a strong sense of purpose, then setting high standards and expectations for achievement; and (iv) *idealized influence*, i.e., modeling ethical and socially desirable behavior and a passion toward work goals.

We additionally considered the concepts of *mindset* and the *decision-making environment*. *Mindset* is a cognitive belief system consisting of interrelated beliefs, assumptions, and knowledge that we use to process information, inform our decisions, and guide our behavior. In addition to *Entrepreneurial Alertness* (Tang and Kacmar, 2010), we took inspiration from the *Entrepreneurial Mindset* (NFTE, 2020), which can be defined as a person who “…recognizes an otherwise overlooked opportunity, develops the confidence to take a risk, communicates their ideas clearly, and is able to adjust to and learn from setbacks” (NFTE, 2020). We were particularly drawn to the Entrepreneurial Mindset when considering the typical academic work environment, which entails frequently developing new research ideas, deciding what to work on and when, and mentoring teams with diverse backgrounds. That is, an academic commonly serves as their own boss, typically existing without any form of instructional manual, having to think on their feet and make sense of dynamic and often uncertain environments. As such, we believe *mindset* to be an integral component of leadership and to the growth and happiness of your team.

The *decision-making environment* encompasses the information available, knowledge of the situation, nature of the problem, the risk associated with the decision, and the *culture* within which the decision is being made (Render et al., 2017). *Culture* refers to the values, beliefs, and assumptions shared by members of the organization. While *culture* is crucial to how things are done, it is elusive because it is anchored in unspoken behaviors, mindsets, and social patterns (Groysbrg et al., 2018). The cruciality of *culture* is especially important to academia, since it shapes how networks are formed, resources are shared and, like it or not, may be important to the tenure process.

## Sciducio framework

**Figure 2:**
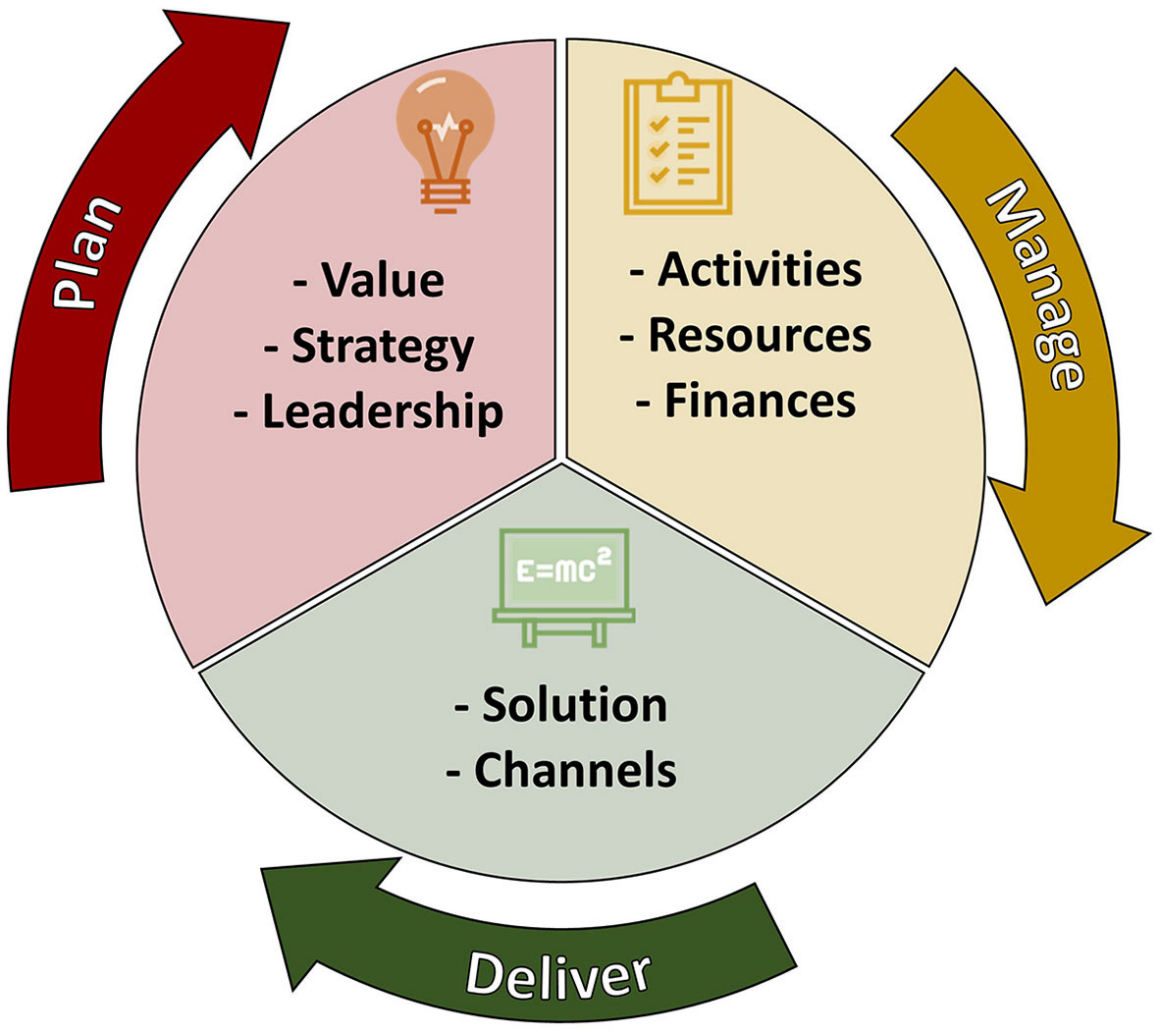
A conceptual model of Sciducio. The inner circle is divided into three parts, with each division representing a Domain (Red: Plan; Amber: Manage; Green: Deliver). The Domains are illustrated via the arrows orbiting the inner circle. The arrows are also numbered from 1 to 3 to indicate directionality. The text inside each division of the circle illustrates the Blocks (Plan: Value, Strategy, Leadership; Manage: Activities, Key Resources, Finances, Deliver: Solution, and Channels) that are used to support each Domain.

The proposed framework is presented in Figure 2 and discussed in more detail below. We elected to define Sciducio as a framework rather than a model or method (Nilsen, 2015). A model can be defined as a simplified approach, often in diagrammatic form, for describing an existing or future state or situation. A method refers to a systematic approach for achieving specific goal(s). A framework lies between a model and a method and consists of a simplified structure or system for the realization of defined goal(s), often containing more than one model. Using the theory discussed in the previous section, and illustrated in Figure 2, Sciducio incorporates three domains (Plan, Manage, and Deliver), eight blocks, and is intended to fit on one-sheet. Table 2 presents example tools that are discussed below and may help to plan and complete each block.

**Table 2:** Tools that may be used to plan and complete Sciducio blocks.

**Tool**	**Description**
Asana	An online project management platform
Gantt chart	Used to display activities against time
Individual Development plan (IDP)	Used to consolidate available resources, identifying individual goals, then plan key opportunities for growth
Lean philosophy	Emphasizes the removal of waste within a process
Microsoft teams	An online, collaborative workspace
Needs analysis	The process of identification and evaluation of needs
Needs assessment	A process to identify skills or knowledge gaps
RACI chart	Used in project management to map task roles and responsibilities
Requirement analysis	A process of identifying, analyzing, and managing project requirements
Strengths, weaknesses, opportunities and threats (SWOT) analysis	A realistic, fact-based, data-driven look at the strengths and weaknesses of an organization

Note that these tools are examples to get you started, other approaches are available and may be suitable for your particular environment.

### Domain: plan

The Plan domain of Sciducio can be likened to the background and objective sections of a research project or manuscript. That is, this domain considers the problem(s) that need to be solved, context for said problem(s), and strategies for tackling said problem(s).

#### Plan block: value

This block broadly fits with the *problem* and *unfair advantage* blocks of the lean canvas (Maurya, 2022) and the *value proposition* and *customer segment* blocks of both the Business Model Canvas (Osterwalder and Pigneur, 2010) and Lean Canvas (Maurya, 2022). The *value proposition* block refers to the product and services the business plans to offer the customer and the *customer segments* block refers to the target audience. The *problem* block refers to the problem that the customer segment has that the business will address, and *unfair advantage* pertains to the business' competitive edge or point or difference. This can include advantages sourced from the team, technology, resources, etc.

The *Value* block can be used to address the following questions:

- *Problem*: what does the group wish to accomplish? What does success mean? The focus does not need to be limited to scientific research. The goals could include, but are not limited to, using science to address defined societal problem(s), improving outcomes for specific populations, community service, and providing mentorship opportunities. Work to understand the problem from the perspective of those who experience it (end-user or beneficiary) or understand it well (scholars).
- *Target audience*: if we solve the problem, who will value this information (narrow and wide focus)?
- *Value proposition*: if we solve the problem(s) what does the final product look like?
- *Unfair advantage*: What is your unique scientific identity? What advantages does this this group possess that are uncommon to the scientific community, and will help this group stand out and be successful? Unfair advantage can come from one or more aspects of the model (e.g., the team, proprietary technology or methodology, resources, partnerships).

Strategies that can assist in addressing the questions posed immediately above include:

- *Problem*: using a white board or online software such as Miro (2022), perform a Strengths, Weaknesses, Opportunities and Threats (SWOT) analysis (Zhukova, 2021).
- *Target audience:* identify the key population or end-user. Narrowing your focus will assist in developing confidence in your chosen area and increase your likelihood of meaningful impact.
- *Value proposition:* don't just identify the problem, think about the “so what?” Practice describing your end-product—–your sales pitch to journals and funding bodies!
- *Unfair advantage*: Resist conformity (Seals, 2022a). Listen to and learn from established investigators, and respect established cultural norms. However, when someone informs you that “this is the way things are done,” do not be afraid to ask why, and do not be afraid to think outside the box and to establish your own brand! (Tregoning and McDermott, 2020; Seals, 2022b). As described above, the SWOT analysis is a useful tool (Zhukova, 2021).

#### Plan block: strategy

For this block, as described in the Theoretical Overview: Strategic Plan section, we took inspiration from widely used components of a business strategic plan (Viardot, 2017).

The Strategy block can be used to address the following questions:

- *Mission statement*: what does this group do? What are the main functions of the group?
- *Vision statement*: if we achieve the mission, what difference will we make?
- *Value statement*: what values best define how each member is expected to conduct themselves in the group?
- *Focus areas*: what, specifically, should we focus on to work toward the vision?
- *Objectives*: with respect to our focus areas, what targets should we set?
- *Projects*: what specific activities will help to achieve the objectives?
- *KPIs*: what should we measure to indicate whether the objectives are effectively being met?

Strategies that can assist in addressing the questions posed immediately above include:

- *Mission statement*: succinctly and clearly outline a clear purpose for the research group existing.
- *Vision statement*: describe the “so what?” i.e., the difference that will result from achieving the mission. Vision can help obtain buy-in from team members.
- *Value statement*: clearly outline the values of greatest importance to the group. These values can be broad and consist of one or two words for each value, e.g., open communication, community, and/or growth mindset.
- *Focus areas*: establish high-priority areas that are more specific than the vision.
- *Objectives*: establish specific, measurable targets, e.g., complete XXX project by XXX.
- *Projects*: establish projects that will specifically help to achieve the objectives, e.g., write a publication on XXX by XXX.
- *KPIs*: establish specific measures of success, e.g., number graduations, publication count, and/or revenue generated (Kerzner, 2017).

#### Plan block: leadership

For this block, as described in the Theoretical Overview: Strategic Plan section, we took inspiration from the Four I's of Transformational Leadership (Northouse, 2021), the Entrepreneurial Mindset (NFTE, 2020), and considered the *decision-making environment*.

The Leadership block can be used to address the following questions:

- *Intellectual stimulation*: how can the “fear factor” be removed to encourage challenges to the status quo?
- *Individual consideration*: what is the vision and what strategies can be used to ensure it is “shared”?
- *Inspirational motivation*: what strategies can be used to ensure team members have a strong sense of purpose?
- *Idealized influence*: what values should be communicated to model ethical and socially desirable behaviors?
- *Mindset*: how is an entrepreneurial mindset fostered, i.e., to identify opportunities, take educated risks, communicate ideas clearly, and learn from setbacks?
- *Decision-making environment*: how can I better understand the culture of the department and larger organization? What are the preferred means and styles of communication? Why is it important to understand the culture?

Strategies that can assist in addressing the questions posed immediately above include:

- *Intellectual stimulation:* in team meetings, ask mentees to challenge assumptions about the way things are always done. Also, empower your team by asking for the thoughts and ideas of all members, and encourage communication between mentees at different career stages.
- *Individual consideration*: keep open lines of communication, recognize individual contributions, and ensure the professional development plan attends to the needs of the individual (Maestre, 2019).
- *Inspirational motivation*: develop a shared culture that bonds the group (Tregoning and McDermott, 2020). This could include identifying a group name, designing a website, and branding activities such as developing a logo and a branded presentation template. You can also use a RACI (Responsible, Accountable, Consulted, Informed) chart (Kendrick, 2013) to delegate key roles and responsibilities. Such responsibilities may include leading social media efforts, journals, clubs, or even lab meetings.
- *Idealized influence:* model ethical and socially desirable behaviors and exhibit enthusiasm about the organization's strategy.
- *Mindset*: part of this can be accomplished through readings [e.g., Growth Mindset: The New Psychology of Success (Dweck, 2006)] and group discussion. Additionally, practice setting clear objectives, decisiveness, redefining failure as an opportunity to grow, facing fears (e.g., public speaking), and actively encouraging curiosity (Master Class, 2022).
- *Decision-making environment*: as part of your appointment, you may be assigned mentors who are senior faculty within and/or outside your department. If you have not been assigned mentors, you may wish to seek such individuals yourself. These networks will be instrumental.

### Domain: manage

The Manage domain of Sciducio can be likened to the methods section of a research project. That is, a transparent recipe for achieving the mission. However, unlike a methods section, management is dynamic and should evolve as the needs of you and your team change. This helps clearly answer the question, “How can we achieve the mission and vision we have collaboratively chartered?”

#### Manage block: key activities

This block, which broadly fits with the *key activities* block of the Business Model Canvas (Osterwalder and Pigneur, 2010), refers to the most important activities in executing a company's value proposition. Key activities represent those things an organization has to do exceptionally well in order to fulfill their value proposition. The most obvious activity to manage is research, though professional development should arguably have an equal or greater footing. Within the academy your team is likely to be comprised of individuals who are there to be formally educated. Below we also consider the environment, administration, and time—each of which are essential to the efficient and effective completion of all activities.

The Key Activities block can be used to address the following questions:

- *Research*: what resources are available to assist with project management?
- *Professional Development*: how do I plan professional development activities and keep each individual engaged?
- *Environment*: what processes do we wish to implement to ensure the environment is most conducive to the planned activities?
- *Administrative:* how do I obtain electronic credentials, learn about safety issues (e.g., ethical review), build an online profile, make purchases and recruit/hire?
- *Time*: how do I schedule my time, and the time of the group, to ensure efficiency?

Strategies that can assist in addressing the questions posed immediately above include:

- *Research*: several project management tools are available, including Microsoft Teams (Microsoft, 2022) and Asana (2022a). To map task roles and responsibilities, use Excel on an online platform such as Teamgantt (Harned, 2022) to build a RACI chart (Kendrick, 2013).
- *Professional Development*: a Needs Analysis (Watkins et al., 2012; Bouchrika, 2022) can be used to identify opportunities for growth and an Individual Development Plan (IDP) to consolidate available resources, identify individual goals, and plan and prioritize key opportunities for growth (Vanderford et al., 2018; Seals, 2022a).
- *Environment*: with respect to the research environment, *Lean Philosophy* can be used to optimize workflow (Womack and Jones, 2003). This includes placing the right things in the right place at the right time and in the right quantity to minimize waste, e.g., for a given project, place blood collection consumables in the most accessible place, the sharps container within easy reach, and ensure an obvious, unobstructed route to the site of blood processing.
- *Administration*: when preparing to seek answers to the questions outlined above (and likely many others!), a checklist may be useful (McKinley et al., 2022). Then, to facilitate ongoing management, utilize a RACI chart (Kendrick, 2013; Hobin et al., 2014).
- *Time*: time is our most precious commodity (Haynes et al., 2006). Try building a Gantt chart within Asana (2022b) or other open-source software (Rebiere and Rebiere, 2017; Goldstein and Avasthi, 2021). Identify periods of the day when tasks must be performed (e.g., teaching, research, service) and by whom. Outside of the must-do tasks, identify the period they are most “switched on,” organizing the most challenging and/or rewarding tasks around this block, then schedule tasks that are less challenging during low-energy periods (typically mid-afternoon) (Martin, 2011).

#### Manage block: key resources

This block broadly fits within the *key resources* and *Partner Networks* blocks of the Business Model Canvas (Osterwalder and Pigneur, 2010) *key resources* can be human, financial, physical, and intellectual, and *partner network* refers to strategic alliances. With respect to Sciducio, we have elected to discuss infrastructure, equipment, people, and networks.

The *Key Resources* block can be used to address the following questions:

- *Infrastructure*: what pre-existing infrastructure is available to support the key activities, e.g., laboratory space, administrative staff, funding mechanisms, etc.?
- *Equipment*: what equipment is required to complete the key activities? What are the priority vs. non-priority equipment items?
- *People*: what type of expertise is required to compliment the team? How many people are required? How do I recruit the right people?
- *Partner Networks*: who do I need to reach out to support our *key activities*?

Strategies that can assist in addressing the questions posed immediately above include:

- *Infrastructure*: audit the environment, seek mentorship, and attend info and training sessions offered by your institution (Goldstein and Avasthi, 2021).
- *Equipment*: conduct a Requirements Analysis (Hay, 2003; Goldstein and Avasthi, 2021; Liston and Lesage, 2021) to better gauge the resources that will be required to effectively complete the *key activities*.
- *People*: conduct a Needs Assessment to identify required tasks and goals, experience level and function (Watkins et al., 2012; Liston and Lesage, 2021; Bouchrika, 2022).
- *Partner Networks*: start by identifying key societies, conferences, on campus activities and other outlets that offer structured networking opportunities (Schweitzer et al., 2019). Foster an environment within your group that encourages actively seeking out and communicating with potential collaborators. Additionally, invite potential collaborators to give a talk either in-person or virtually.

#### Manage block: finances

This block broadly fits with the *fixed costs* and *variable costs* blocks of the Business Model Canvas (Osterwalder and Pigneur, 2010), the *Cost Structure* of Lean Canvas (Maurya, 2022), and Revenue Streams of both models. *Fixed costs* are unchanged across applications (e.g., salary or rent), *Variable costs* vary depending on the costs required to produce and deliver goods or services, and *cost structure* can be considered an amalgamation of fixed and variable costs. *Revenue streams* pertains to how you intend to generate money to support the fixed and variable costs.

The *Finances* block can be used to address the following questions:

- *Fixed Costs*: what are the minimum costs required to keep the research environment operating, e.g., basic supplies, salaries, assistantships, professional development, etc.?
- *Variable Costs*: what are the costs associated with specific key activities, e.g., research project, publication, etc.?
- *Revenue Streams*: what revenue streams are readily available to support baseline costs, e.g., department-supported laboratory budget, assistantships etc.? What revenue stream can I seek to support specific research projects, e.g., internal, federal, foundation or industry grants.?

Strategies that can assist in addressing the questions posed immediately above include:

- *Fixed Costs*: speak with administrative staff within and outside your department to comprehend available resources and budget running costs.
- *Variable Costs*: for funding applications, your institution will likely make available budget templates that include line items and calculate indirect costs.
- *Revenue Streams*: familiarize yourself with your office of sponsored programs to learn about internal and external funding opportunities. Additionally, many funding agencies provide email postings about upcoming opportunities. Using these sources of information, develop a [dynamic] timeline and plan for funding opportunities to apply to Seals (2021).

### Domain: deliver

The *Deliver* domain of Sciducio can be likened to the results and conclusion sections of a research project. We have purposefully omitted discussion from the comparison to a research problem. A well written research paper includes logical, concise results which are devoid of interpretation, and a conclusion that completes the story arch by specifically relating the final product or solution back to the problem. Note that we did not include grant funding within this section. Our belief is that funding should serve as a means to an end, to facilitate a solution to a real problem, it is not the end-product.

#### Deliver block: solution

This block broadly fits within the *customer relationship* block of the Business Model Canvas (Osterwalder and Pigneur, 2010) and the Lean Canvas (Maurya, 2022) and the *solution* block of the Lean Canvas (Maurya, 2022). The *solution* block is intended to be a concise and specific statement pertaining to the *minimal viable product*—the intended final product. *Customer relationship* refers to the target population, i.e., to what or whom will the solution be useful. Within the academy, our target is research, teaching, and service.

The *Solution* block can be used to address the following questions:

- *Research*: what difference do you wish to make through your research?
- *Education*: what does a successful mentee graduating from your team look like?
- *Service*: using your research and teaching experience, what novel contributions can you make to the community and to your field?
- *Impact*: through the above solutions, what is the anticipated impact?

Strategies that can assist in addressing the questions posed immediately above include:

- *Research:* when planning specific research projects, make it common practice to consider how/why the outcomes from the research will be applied. This doesn't necessarily mean immediate public impact—A discrete project may be important to your long-term research agenda.
- *Education*: there is not a singular mentorship approach that will suit all mentees. However, an IDP can provide a structured approach to mentorship that aligns with the *Value* block.
- *Service*: seek opportunities to engage with underserved populations, identify and give public talks, and become involved in societies within your field. However, if you are an early-stage faculty member, do not be afraid to protect your time (Rebiere and Rebiere, 2017; Goldstein and Avasthi, 2021). Focus on currencies of success (Seals, 2022a)—Opportunities that will benefit your career and through which you can have meaningful impact.
- *Impact*: start with your *Vision Statement*, but then consider your specific research, education, and service activities.

#### Deliver block: channels

This block broadly fits with the *Channels* block of the Business Model Canvas (Osterwalder and Pigneur, 2010) and the Lean Canvas (Maurya, 2022). *Channels* pertains to the channels (e.g., physical store, website, or distributors) through which a company can deliver its *value proposition* to the *target customers*. This is likened to the outlets or veins whereby products and services can reach through to customers. From an academic perspective this can be thought of as opportunities for dissemination (e.g., publications, conference presentations, community outreach). In the context of Sciducio, we refer to the *channels* through which the solution will be delivered.

The Channels block can be used to address the following questions:

- *Publications*: through which outlets can your research findings be disseminated?
- *Practice*: how can you directly influence current practices in your field and/or communities?
- *Public*: what avenues can you explore to help your research endeavors have greater appeal?

Strategies that can assist in addressing the questions posed immediately above include:

- *Publications*: focus on identifying journals that will reach your *target audience*—–and write to that audience. Also, develop writing strategies that will strengthen the quality of your prose and get you cited [tip: read Joshua Schimel's Writing Science: How to Write Papers That Get Cited and Proposals That Get Funded (Schimel, 2011)]. Citation count and the impact of the published works are far more important than publication number—but this requires both high quality science and high-quality writing.
- *Practice*: when designing research, have a clear goal in mind with respect to how the findings will be disseminated and utilized.
- *Public*: identify suitable public forums, including public speaking and social Media outlets, e.g., use Twitter to disseminate research findings in a manner that your target audience (i.e., the population impacted by the research) can consume.

## Instruction for use

**Figure 3:**
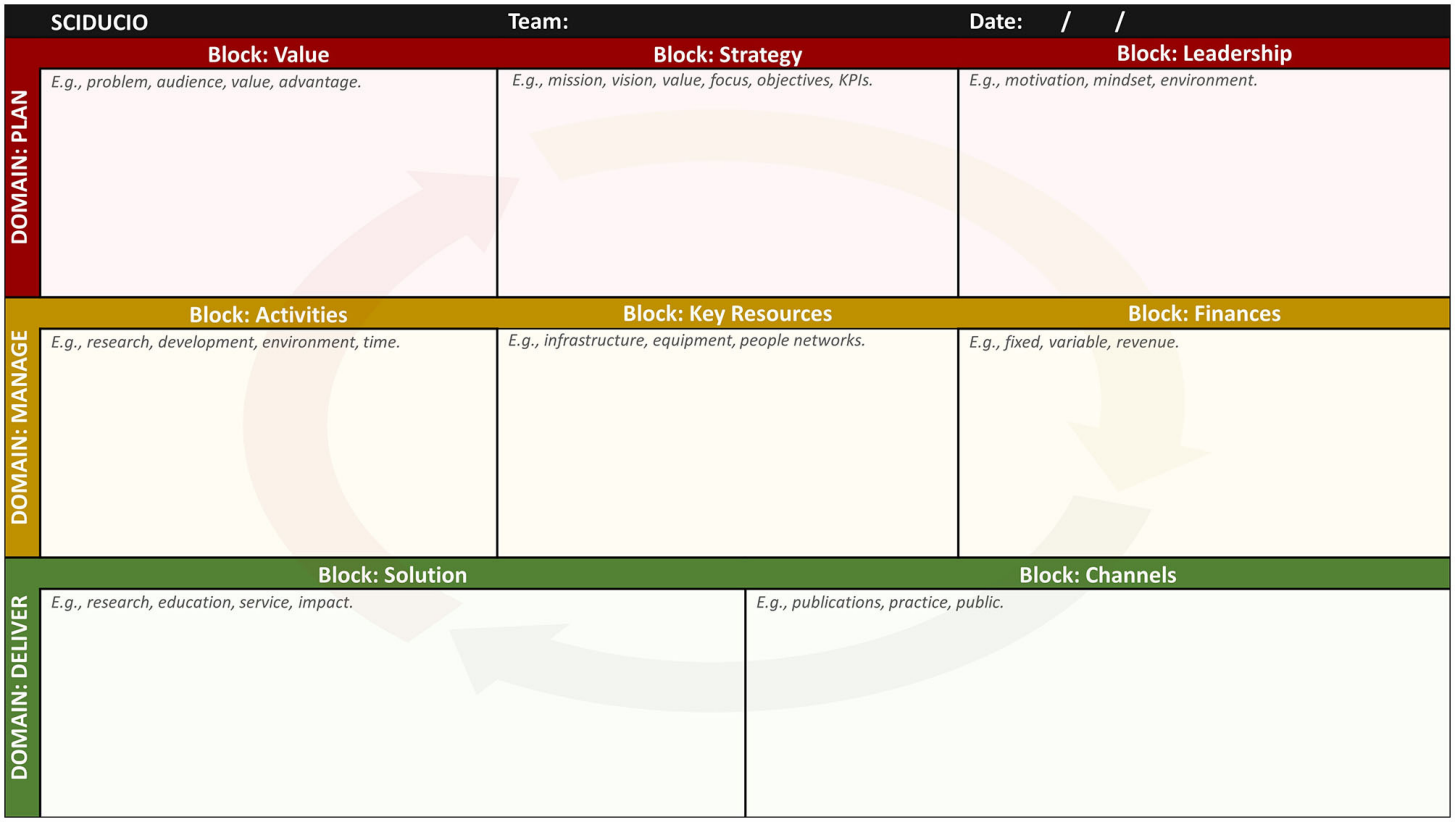
The Sciducio Canvas, incorporating three Domains and eight Blocks (Value, Strategy, Leadership, Activities, Key Resources, Finances, Solution, and Channels). The text inside refers to recommended questions for guide discussion. These questions are essential for getting discussions started and for guiding the process, directions, and decisions made.

**Figure 4:**
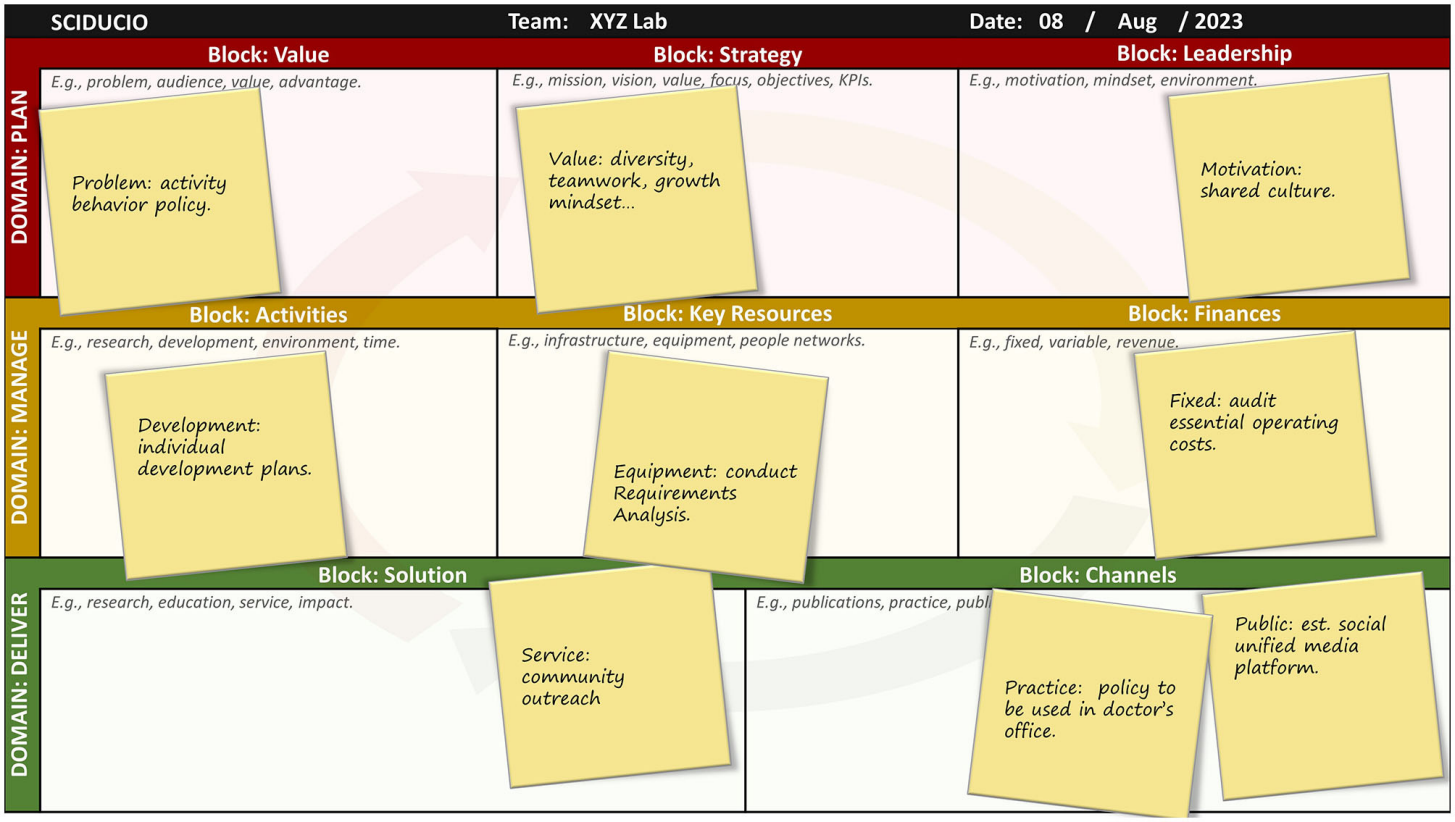
An example of a partially complete Sciducio Canvas. This example indicates how sticky notes may be used. We recommend holding a collaborative brainstorming session and provide sticky notes to each team member. Encourage each team member to write down their ideas related to each block of the Sciducio Canvas. We recommended writing the ideas in short-hand to ensure the populated canvas is digestible. As the canvas fills up, identify patterns, prioritize ideas, and develop action plans based on the insights gained.

A Sciducio template is provided in Figure 3, with a partially completed example presented in Figure 4. Utilize this template in concert with the steps outlined below. As a new faculty member, you may wish to sit down with the one-sheet Sciducio template, either physical or electronic, and use it as a platform to identify and map key considerations for your new post. In this sense, Sciducio is a planning guide for helping you codify your goals, organize your thoughts, frame your team, and chart a course toward success. As an established faculty member, who is either changing position or staying put, Sciducio can be used as a vehicle to organize the multiple facets of academic life, and/or to plan a new direction. As a doctoral or postdoctoral mentee, Sciducio can be used as a training tool, to identify and learn about life as an academic. Do remember that Sciducio is intended to be descriptive rather than prescriptive. The blocks are intentionally designed to be broad, to permit freedom of thought. There is no one-size-fits-all model for success, and nobody understands the inner-workings and goals of your team better than you and your team. Also, Sciducio is intended to be a dynamic, transparent, living document that will facilitate reflection and incremental progression.

While an extensive how to guide is outside the scope of this conceptual framing document, the practical application of this framework is an important area for further discussion. The following steps are recommended as a preliminary departure point for utilizing the Sciducio framework. This is not intended to be a prescriptive series of steps. These are initial suggestions for framing the larger conversation around goals, resources, operations, and skill sets needed to guide thinking and actions.

1. **Step 1:** With a small team, meet and discuss the Sciducio framework. Ensure all team members are familiar with the Sciducio concept and its purpose. Discuss the three domains (Plan, Manage, and Deliver) and the eight blocks (Value, Strategy, Leadership, Activities, Key Resources, Finances, Solution, and Channels) with the team.
2. **Step 2:** Create a copy of the Sciducio Canvas. On the Canvas page of the Sciducio website (sciducio.thecml.net) you will find Jamboard, PDF and PowerPoint formats. All of the formats have been designed to work digitally. We recommend the Jamboard format and displaying on a large screen monitor/TV (though Jamboard does permit real-time collaboration via individual computers/tablets). Alternatively, print the PowerPoint version as a poster Ensure the text and blocks are legible when viewed at the desired distance.
3. **Step 3:** Define the objectives of the strategic planning session. Communicate what the team aims to achieve through the Sciducio Canvas exercise. Start with a specific focus area, such as a new project, product, or process improvement. Utilize the recommended questions for each of the blocks to guide discussion. These questions are essential for getting discussions started and for guiding the process, directions, and decisions made. If you wish to keep things simple to start with, we recommend focusing first on the Problem, Key Activities, and Solution blocks.
4. **Step 4:** Using the digital sticky note feature on Jamboard, or actual/physical sticky notes, encourage each team member to brainstorm ideas related to each block of the Sciducio Canvas. We recommended writing the ideas in short-hand, focusing on the major concepts rather than specific details. Limiting verbosity will lead to the development of a more lucid and digestible framework.
5. **Step 5:** Once the brainstorming session is complete ask team members to share their ideas and insights. Focusing on one block at a time, arrange the sticky notes within the block to capture relevant information and connections. Throughout this process encourage active participation and discussion within the team. As the canvas fills up, identify patterns, prioritize ideas, and develop action plans based on the insights gained.

Remember that the Sciducio Canvas is a flexible tool, and the team can adapt and modify it to suit their specific needs. Regularly review and update the canvas as the team progresses in their strategic planning journey.

## Conclusions

The intent of Sciducio is to provide a practical framework for guiding the development and leadership of an academic research environment. Sciducio incorporates three domains, encompasses eight blocks, and is intended to fit on one-sheet. The Plan domain includes the blocks: *value, strategy*, and *leadership*. The Manage domain includes the blocks: *activities, key resources*, and *finances*. The Deliver domain includes the blocks: *solution* and *channels*. Considering each of the framework blocks is complex, we cannot provide full justice to each component. This paper serves as a general overview and subsequent papers will be more topic specific. Additionally, we encourage others to contribute to this framework.

## Data availability statement

The original contributions presented in the study are included in the article/supplementary material, further inquiries can be directed to the corresponding author.

## Author contributions

LS: conceptualization, methodology, resources, writing—original draft, writing—review and editing, supervision, and product administration. CP, PP, and YL: conceptualization and writing—review and editing. SH and EC: writing—review and editing. LP and NW: conceptualization, literature and theory, and writing—review and editing. All authors contributed to the article and approved the submitted version.
